# Fetal nuchal translucency scan in Nigeria

**DOI:** 10.11604/pamj.2014.18.62.3291

**Published:** 2014-05-18

**Authors:** Olufemi Adebari Oloyede, Mkpe Abbey, Adeniyi Adebowale Oloyede, Onyinye Nwachukwu

**Affiliations:** 1 Fetal Medicine Unit, Olabisi Onabanjo University Teaching Hospital, Sagamu, Ogun State, Nigeria; 2 High Rocks Fetal Medicine and Genetic Diagnosis Centre, Oshodi, Lagos, Nigeria; 3 Whipps Cross University Hospital, London; 4 Manor Hospital, Walsall, United Kingdom

**Keywords:** Nuchal translucency scan, Performance, Down syndrome

## Abstract

**Introduction:**

To evaluate the performance of first trimester nuchal translucency scan screening among pregnant women in Nigeria.

**Methods:**

A prospective observational and questionnaire based study involving 510 pregnant women between 11^+0^ and 13^+6^ weeks. Routine counselling and nuchal translucency measurement was conducted using the FMF, London guidelines. Chorionic villous sampling was done at NT ≥ 2.5 mm or ≥ 95th centile.

**Results:**

Five hundred and ten out of 542 (94.1%) were analysed, mainly referred by health care workers (87.2%) and from predominantly private facilities (94.3%). The number of NT scans performed increased in successive years with corresponding decrease in the mean scanning time. Scan was successfully completed at first attempt in (96.5%), with mean scanning time of 28.3 minutes. Nuchal translucency increases with gestational but not maternal age. The median and 95th centile at 11^+0^ week was 1.2mm and 1.7 mm and at 13^+6^ weeks was 1.5 mm and 2.2 mm. Using a cut-off of ≥ 2.5 mm or ≥ 95th centile, 17 (3.3%) screened positive. Three out of the 17 had invasive testing and 2 (DR = 66.7%) were confirmed trisomy 21, with a false positive rate of 5.9%. Although majority (86.4%) were willing to have invasive testing, only few (3 or 17.6%) of the high risk group had testing.

**Conclusion:**

The study demonstrated that NT scan is feasible as a screening tool in pregnancy in Nigeria. Measures of improving utilization include wider dissemination of information, provision of dedicated NT clinics and manpower training.

## Introduction

Nuchal translucency (NT) has found useful application as an antenatal screening marker for chromosomal abnormalities in the late first trimester of pregnancy [1–3]. As a routine, it provides a first trimester detection rate of about 80% for Down syndrome pregnancies for a false positive rate of about 5%, and even superior detection rates for trisomy 13 and 18 [2–4]. It was introduced into clinical practice in the 1990s, with most of the data derived from researchesconducted and supervised by centres in European centres using mostly Caucasian population, while the derived values are used in all women irrespective of ethnic origin [2–6]. There are controversies about its implementation as a routine screening method especially in low risk population attributable to poor reproducibility of measurement, high false positive rates and poor screening sensitivity [7–9]. Its major advantage however, is that it provides opportunity for an early pregnancy screening and decision about invasive procedures in high risk group. Where a termination of pregnancy is decided after evaluation, it is safer in the first trimester than abortion following second trimester prenatal screening procedures. An additional benefit is that major structural abnormalities can be ruled out at same scan session. The nuchal translucency scan was introduced into clinical practice in Nigeria less than a decade. However, at present there is no data on its performance in the country. Similar to any other new service, its utilization and performance can be affected by numerous factors ranging from manpower, facilities, safety and perceived outcome.

The aim of the present study was to report on the performance of routine nuchal translucency scan, assess its relationship with maternal and gestational age in Nigeria as well as the detection and false positive rates for Down syndrome

## Methods

We studied data from 510 women who attended Fetal Medicine and Genetic diagnosis centre between January 2007 and December 2012 for antenatal screening for Down syndrome using the nuchal translucency scan. The women were made up of heterogenous groups of antenatal patients from both private and public hospitals in Lagos, Nigeria.

The NT was performed by a Fetal Medicine consultant, certified by the Fetal Medicine Foundation, London. All the women received pre- and post-screening counselling and provided informed consent to participate in the study. They all fulfilled the inclusion criteria defined as singleton gestation within 11^+0^ and 13^+6^weeks, when the CRL is between 45mm and 84mm. Excluded were plural pregnancy, foetuses with obvious structural defect and spotting per vaginam. An NT image was considered satisfactory, if the fetus is in the mid sagittal position and the fetal head neck and upper thorax occupies at least three quarter of the film. The callipers are placed on the area of maximum translucency in the cervical region, using the on-to-on rule over the cervical spine [4]. The NT scan was performed using Imagynae, Sonoscape or HDI 1500 with a 3.5-7.5mmHz transabdominal convex transducer. An important feature of the scan machine is that it must measure to 0.1mm and has cine loop video function. The ultrasound scan measurements were dually recorded: astraia software and manually. We defined high risk as NT measurement ≥ 2.5 mm or ≥ 95^th^ centile. The FMF risk cut-off of 1:200 was not used because the licence was not available in some years during the study period. Women in the high risk category were counselled and offered chorionic villous sampling for molecular karyotyping using QF PCR technique.

Each woman filled a questionnaire on her biosocial characteristics, preference for a first trimester screening nuchal translucency scan and desire to have invasive test, if the risk is high. The source of information and referral were also documented. Data were analysed using the EPI info version 6. The range, mean and median values of the NT were determined for gestational age and maternal age and presented as percentages and frequencies.

## Results

During the study period, a total of 542 women had routine NT scan, of which 510 (94.1%) fulfilled the inclusion criteria and were analysed. The mean maternal age was 32.8 ± 2.8 years, with a range of 21 and 47 years. The highest proportion of women was below 35 years (94.1%). The parity ranged between 0 and 6. The majority (79.5%) were less than para 3 (Table 1).

**Table 1:** Maternal Age and Parity distribution

Characteristics	Responses (N = 510)	Percentage (%)
**Ages (years)**		
20-24	31	6.1
25-29	208	40.8
30-34	246	48.2
35-39	25	4.9
**Parity**		
0	201	39.4
1-2	205	40.1
3-4	94	18.5
>5	10	2.0

**Figure 1:**
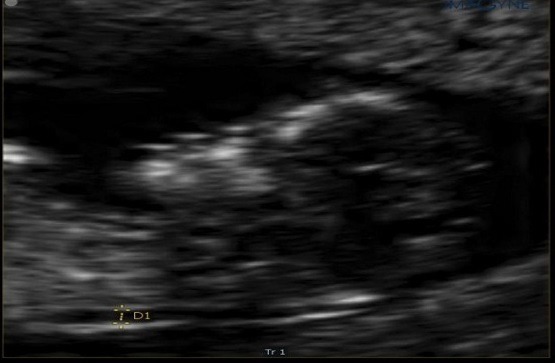
Nuchal translucency image

Figure 1 shows the nuchal translucency scan image according to FMF criteria. Three quarter of image is occupied by the head, neck and upper thorax, with the fetus in midsaggital position and NT measured over the area of maximum translucency using the on to on caliper rule

**Figure 2:**
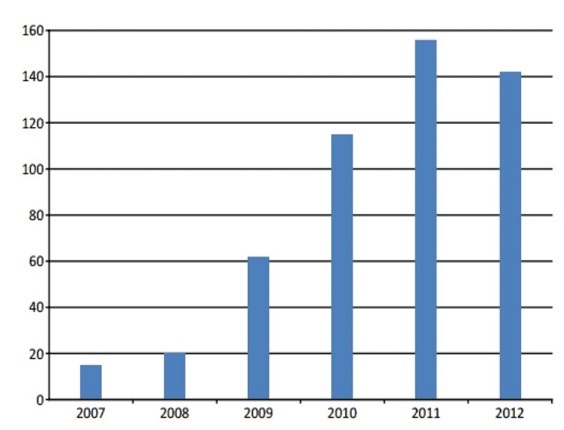
Trend of utilization of procedure

Figure 2 depicts the trend in the utilization of scan, which confirmsincreasing number of procedures in successive years. The number of scans performed was lowest (15 or 2.9%) at inception in 2007 and stepped up drastically to above 100 per year from 2010. The highest was in 2011 (156 or 31.1%)

Nuchal translucency scan was successfully completed within 45 minutes in 492 (96.5%) cases at first session, and the remaining 18 (3.5%) were concluded at a repeat session. With respect to frequencies of repeat per year, it was highest (26.7%) in 2007 and lowest (0.7%) in 2012 (Table 2). Throughout the study period, the mean time to complete the scan was 28.3 minutes with a minimum to maximum interval of 15 - 55 minutes. The scanning time proportionally reduced steadily with increase in the number of scans performed. In 2007, it was 45 minutes and in 2012, it was 23 minutes.

**Table 2:** Success of Nuchal Translucency Scan

Year	Number	First Attempt (%)	Second Attempt (%)	Mean Scanning Time (minutes)
**2007**	15	11 (73.3)	4 (26.7)	45
**2008**	20	16 (80.0)	4 (20.0)	42
**2009**	62	59 (95.2)	3 (4.8)	32
**2010**	115	112 (97.4)	3 (2.6)	30
**2011**	156	153 (98.1)	3 (1.9)	28
**2012**	142	141 (99.3)	1 (0.7)	23

The proportion of women with respect to GA shows that 178 (34.9%) were between 11-11^+6^ weeks, 190 (37.3%) between 12-12^+6^ weeks and 142 (27.8%) were between 13-13^+6^ weeks (Table 3 - A). The median NT and 95th with a range of 1.1-2.6 mm while at 13 weeks, the median NT and 95th centile were 1.5mm and 2.0mm, with a range of 1.2- 2.4mm (Table 3 - A).

**Table 3:** Performance of Nuchal Transparency Scan

A: Relationship between Nuchal Translucency and Gestational/Maternal Age						
Parameters	Number (%) (N= 510)	Nuchal Transparency (mm) Range		Median	Mean	95th Percentile
**Gestational age**						
11 + 0 – 11 + 6	178 (34.9)	1.0 – 2.6		1.2	1.1	1.7
12 + 0 – 12 + 6	190 (37.3)	1.0 – 2.0		1.3	1.3	1.8
13 + 0 – 13 + 6	142 (27.8)	1.2 – 2.4		1.5	1.45	2.2
**Maternal Age**						
21-25	22 (4.3)	1.1 - 1.5		1.2	1.2	1.3
26-30	78 (15.6)	1.2 - 1.6		1.4	1.3	1.4
31-35	244 (48.8)	1.1 - 2.6		1.5	1.3	1.9
36-40	133 (26.6)	1.1 - 1.7		1.4	1.3	1.5
>40	33 (6.6)	1.2 - 1.6		1.4	1.3	1.4
**B: High Risk Determination**						
**NT Cut-off**	**No of Pregnancies**	**Screen positive Rate (%)**	**No of CVS**	**No of Downs Rate (%)**	**Detection Rate (%)**	**False Positive**
2.5 mm/95th centile	17	3.3	3	2	66.6	5.9
> 2.5 mm alone	4	0.8	1	1	100.0	-
> 95th centile alone	13	2.5	2	1	50.0	7.7

Using NT cut off of = 2.5 mm or = 95^th^ centile to define screen positive, 17 pregnancies were classified as high risk, giving a screen positive rate of 3.3%. However only 3 (17.6%) had chorionic villous sampling for molecular karyotyping and of which 2 were confirmed to have Down syndrome from QF PCR technique. This gives a detection rate of 66.7% and a false positive rate of 5.9%. One of them is above 35 years and the other below 35 years of age.

In terms of specifics (Table 3 - B), when NT cut-off of 2.5 mm was used, 4 (Screen positive rate = 0.8%) pregnancies were high risk, 1 had CVS and was confirmed (Detection rate= 100%) as Down syndrome. For NT = 95^th^ centile, 13 (Screen positive rate = 2.5%) were high risk, 2 had invasive testing and 1 (50.0%) of them was Down syndrome.

The source of information was health workers in 145 (87.2%) women, public media in 48 (9.4%) and friends or relatives in 17 (3.4%) for women. The majority (94.3%) was referred from private health institutions, with only 5.7% from public health institutions (Table 4). None of the women had previously affected children. All the women prefer to have first trimester screening in subsequent pregnancies. Concerning willingness to have an invasive testing (Chorionic Villous Sampling) if the risk is high, 441(86.4%) were willing, while 48 (9.0%) were not willing and 23 (4.5%) were undecided. The opinion of the women on the factors that should be addressed to improve on the availability and utilization of NT screening showed that most women would want a wider dissemination of information through the public media 420 (82.4%), more trained personnel and NT clinics 491 (96.3%). There were multiple responses.

**Table 4:** Improving Utilization of Nuchal Translucency Scan

Characteristics	Responses (N = 510)	Percentage (%)
**Information Source**		
Health Worker	445	86.5
Friends/ Relatives	48	9.4
Public Media	17	3.4
Referrals		
Private health facilities	478	98.7
Government health facilities	32	6.3
**Invasive testing**		
Willing	441	86.4
Unwilling	46	9.0
Undecided	23	4.5

## Discussion

Utilization of first trimester nuchal translucency screening is limited in Nigeria. Consequently the feasibility and performance of NT remains to be determined and documented. In Nigeria, it is commonly believed that chromosomal abnormalities are not as prevalent in western countries while there is dearth of trained manpower to undertake genetic counselling, screening and follow up diagnostic procedures when necessary. This study, has demonstrated that it can be implemented in developing countries like Nigeria, similar to the practice in many western countries [3–5]. The first indicator of this assertion is the increasing number of procedures in successive years. The utilization could probably be more than we reported, if NT scan Down syndrome is a routine component of antenatal care services, similar to the practice in the many developed countries [10]. Consequently, manypregnancies are delivered without the benefit of screening, even though studies have shown that majority of women prefer first trimester screening [11, 12]. Also, previous studies in Nigeria have shown the positive influence of improved socioeconomic, education and cultural factors on the utilization of other prenatal diagnosis service in the country [12, 13].

The number of successful scanning at first attempt and reducing scanning time were demonstrations of the learning curve. Our figure (96.5%) is an improvement onsome studies that quoted lower success rates (82%;58% improved to 74% at = 10 weeks gestation) at first attempt [9, 14]. This could be explained by our strict adherence to guidelines for NT measurement especially in respect of gestational age, rigorous training and regular auditing of performance. The reduction in scanning time could also be attributed to improvement in skill of sonologist. Nuchal translucency scan require additional time than required for routine booking scan and both patients and physicians should appreciate this fact when booking for the procedure. Failure of success at first attempt has been attributed to time inadequacy, maternal obesity and inappropriate fetal attitude [15].

The study confirms a direct positive relationship between nuchal translucency values and gestational age, while that between the nuchal translucency and maternal age is non-linearly defined. Similar observation was reported in earlier researches, but contrasts the relationship with maternal age reported by other researchers [9, 11, 16].

The most widespread application of nuchal translucency measurement is the screening for aneupliodies especially Down syndrome. The detection rate obtained in this study based on a cut off NT value of = 2.5 mm or = 95^th^ centile was 66.7%, for a false positive rate of 5.9%. This rate however might not truly represent the pregnant population because only 3(17.6%) of screened positive pregnancies had chorionic villous sampling for confirmation. Study from Taiwan reported similar figures of detection and false positive rates [17]. These figures are within the wide range (33% - 91%) reported by other authors, even though the most quoted is 76%-81%[15, 17–19]. Several factors could be responsible for this observation. First, the cut-off parameter to define high risk has not been uniform in many studies. The more recent cut-off is based on a risk of 1:200 pregnancies based on combination of Maternal age, NT and other secondary markers as proposed in the FMF software. We are unable to use this parameter because the licence to use the software was not available for the entire duration of the study. This is one of the challenges in developing countries like Nigeria. We also think that differences in the obstetric characteristics and small study population could contribute to the difference in the rates. Consequently, these factors must be addressed in order to produce rates that can be compared with figures from developed countries. When a fixed cut-off value of 2.5 mm was used to define high risk, the detection rate was 100% and 0% false positive rate. This must however be taken with caution because NT has been shown to increase with gestational age and by implication the risk for chromosomal abnormality. It is therefore theoretically unlikely to get a 100% detection using a fixed value for all gestational ages. Better performance has been reported using gestational age related cut-off as was done in some pregnancies in this series [17, 20]. Although the detection and false positive rates differ from values obtained using 2.5mm, they are however still close to the widely quoted figures.

Our study has again confirmed that health workers are the highest source of information and private health facilities remain the major source of referrals as previously reported in a study in similar local population [10]. In order to achieve greater success, it is important to provide additional education to these groups, while encouraging a review of the antenatal practice in public facilities. There is widespread public speculation that the utilization of invasive procedures would be extremely low because of religious believe about ‘unravelling Gods work’. These believe is perceived to be a possible explanation for the negative attitude of women towards invasive testing from this study. While the questionnaire reveals more women willing to have chorionic villous sampling, the eventual number that had the procedure was significantly lower. The lesson is that women's decision before screening cannot be relied upon as the final decision when the outcome is known. Hence the importance of good post procedure counselling. The public media being a strong means of communication is recommended for wider dissemination as found in the present study

## Conclusion

The two important limitations of the study are the low study population and cut-off parameter. To use 1:200 as cut-off, can be easier when NT becomes more wide spread and licence are regularly updated. In spite of these limitations, the study has provided the first report showing that NT scan is feasible as a potential screening tool in early pregnancy, applicable in developing countries such as Nigeria. Gestational age related cut-off give a better performance than a fixed cut-off, while individual risk of > 1:200 using the FMF software is recommended for optimum performance. Training, wider information dissemination and dedicated NT scan screening clinics improve utilization. Further data in future shall address some of the constraints identified in this study.
